# Biofabrication in suspension media—a decade of advances

**DOI:** 10.1088/1758-5090/addc42

**Published:** 2025-06-03

**Authors:** Megan E Cooke, Morgan B Riffe, Manuela E Gomes, Rui M A Domingues, Jason A Burdick

**Affiliations:** 1 BioFrontiers Institute, University of Colorado Boulder, Boulder CO 80303, United States of America; 2 Materials Science & Engineering, University of Colorado Boulder, Boulder CO 80303, United States of America; 3 School of Medicine and Biomedical Sciences (ICBAS), Unit for Multidisciplinary Research in Biomedicine (UMIB), University of Porto, Porto 4050-313, Portugal; 4 INL – International Iberian Nanotechnology Laboratory, Braga 4715-330, Portugal; 5 Department of Chemical and Biological Engineering, University of Colorado Boulder, Boulder CO 80303, United States of America

**Keywords:** suspension bath, bioprinting, bioinks, embedded printing

## Abstract

Suspension bath bioprinting, defined as extrusion bioprinting into a suspension bath consisting of a yield-stress material with fast recovery, emerged over a decade ago. Since this time, many suspension baths have been developed from molecular assemblies to granular media and across a range of synthetic and natural polymers. These suspension baths have been applied to the printing of a wide variety of inks for applications in tissue engineering, from *in vitro* tissue models to implantable constructs. In a scoping search of published literature over the past decade, 254 articles were identified that met various definitions related to suspension baths for biofabrication in order to gain a perspective on the various materials used and their applications; however, the literature is much more broad than this due to the disperse terminology that has been applied to the approach. This article gives a perspective on the progress that has been made in suspension bath printing, including applications of the technology and challenges that exist across the field, as well as provides a look to the future of where such printing methods will make an impact.

## 1. Introduction: early developments in suspension bath bioprinting technology

**Figure 1.**
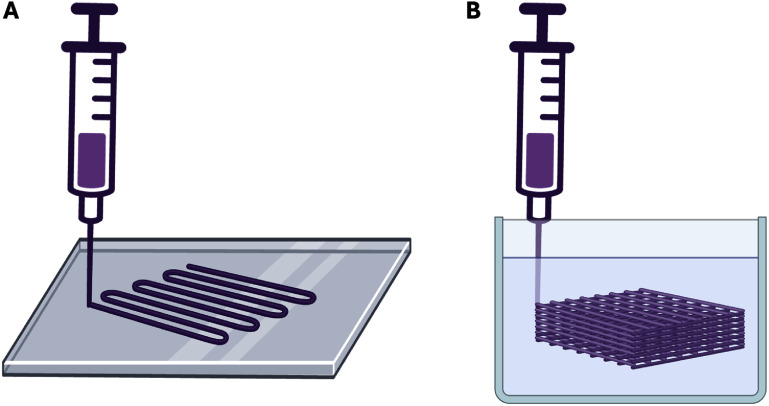
Schematic illustration of extrusion bioprinting. (A) Traditional direct extrusion bioprinting involves the deposition of material onto a substrate in air, which then proceeds in a layer-by-layer manner. (B) Suspension bath bioprinting involves the extrusion of a material within a reservoir of a yield-stress material termed a ‘suspension bath’ that rapidly recovers once the material is deposited.

Bioprinting strategies emerged as early as 1988 under the term ‘cytoscribing’, which described the precise spatial deposition of cell-seeded materials [1]. As the field of biofabrication progressed from droplets to multi-layered structures, materials traditionally used in tissue engineering such as alginate, gelatin, and hyaluronic acid were then adopted into extrusion bioprinting approaches (figure 1(A)). To ensure good print fidelity in the production of multi-layer prints, it was necessary to keep the viscosity of the bioink solution relatively high. This, however, limited the printing of desirable soft materials and was shown to negatively impact cell viability due to the shear stresses experienced by cells during extrusion [2]. Further, printing was restricted to a layer-by-layer approach, which prevented the bioprinting of many complex structures. Over a decade ago, suspension bath bioprinting was developed to overcome these limitations, as it enables the printing of low viscosity biomaterials into complex structures with nearly complete geometric freedom. Specifically, suspension bath bioprinting uses a reservoir of material (suspension bath) that is often a yield-stress hydrogel to hold the bioink in place for subsequent crosslinking or solidification (figure 1(B)) [3]. This approach has now addressed many challenges, such as the printing of cell-only materials and the production of intricate vascular networks, which has led to significant advances in the field of tissue engineering from *in vitro* models to transplantable tissues [4–6].

**Figure 2.**
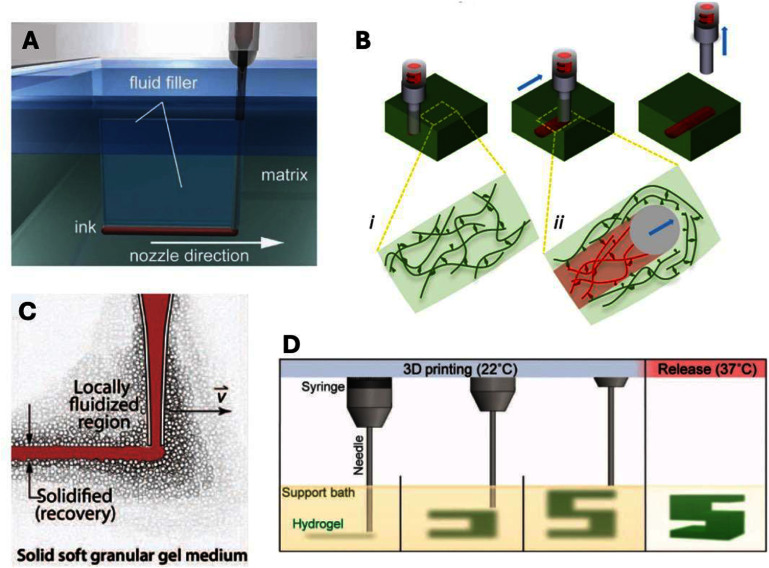
Early suspension bath bioprinting methods. (A) Omnidirectional printing: material extruded into a Pluronic matrix with a reservoir of liquid used to backfill and stabilize the printed part [8]. John Wiley & Sons. [Copyright © 2011 WILEY-VCH Verlag GmbH & Co. KGaA, Weinheim]. (B) Guest-Host printing: *β*-cyclodextrin and adamantane modified hyaluronic acid form non-covalent bonds that can be dissociated with the application of shear and quickly reform when shear is removed [11]. John Wiley & Sons. [© 2015 WILEY-VCH Verlag GmbH & Co. KGaA, Weinheim]. (C) Granular media: Carbopol is a soft granular gel that is locally fluidized with the application of shear and recovers quickly to form a solid-like structure. From [12]. Reprinted with permission from AAAS. (D) FRESH printing: a slurry of fractured gelatin is deformed with shear and recovers quickly into a solid-structure that can then be heated into its liquid state for the removal of the printed part. Reproduced from [13]. CC BY 4.0.

The term ‘suspension bath bioprinting’ encompasses techniques that use automated control systems to deposit materials into a suspension bath, which is defined here as ‘*a yield-stress fluid capable of shear recovery to support printed material that is deposited within its volume*’. Either the suspension bath or deposited material must resemble features of a bioink, defined as ‘*a cell-containing formulation that is processable with 3D-bioprinting techniques*’ [7], although there are many examples of this printing approach being used with only acellular materials. The first example of a suspension bath-like system was published by Wu *et al* in 2011 under the term ‘omnidirectional printing’. This study used a Pluronic bath with a liquid layer on top, such that the bath was back-filled after being sheared by a needle [8]. While not technically a true suspension bath based on the yield and recovery profile, it was an early example of this type of extrusion bioprinting within the volume of another material (figure 2(A)). Shortly after, in 2013, work by the Fisher lab investigated the use of high-density fluorocarbons as hydrophobic baths for ‘submerged bioprinting’. Inspired by early droplet-based bioprinting, these studies deposited droplets of cell-seeded agarose into perfluorotributylamine or Fluorinert (3M) baths, to form complex geometries, including bifurcating tubular structures [9, 10].

Subsequently, in 2015, a flurry of studies utilizing bioprinting into yield-stress baths with excellent shear recovery were published. For example, Highley *et al* utilized assemblies of hyaluronic acid modified with guest-host complexes of adamantane and *β*-cyclodextrin as both a suspension bath and ink. The non-covalent and reversible bonds between adamantane and cyclodextrin rendered this material shear-thinning with fast recovery of structure when the applied stress was removed (figure 2(B)). Bhattacharjee *et al* used a Carbopol granular bath as a suspension bath for printing. Carbopol exists as small polyacrylic acid-based particles that have been widely used as baths for printing as well as to provide data for computational models due to their overall consistency uniformity (figure 2(C)). Lastly, Hinton *et al* reported on freeform reversible embedding of suspended hydrogels (FRESH), which utilized a fractured gelatin slurry as its bath. This has become a widely adopted suspension bath that is ‘reversed’ by heating to 37 °C, liquefying the gelatin and enabling the extraction of the printed structure (figure 2(D)). In the years following, many more strategies have been presented, including the use of agarose fluid gels [14], laponite nano-clay suspensions [15], gellan fluid gels [16], and microgels [17]. It should also be noted that either the bath or the bioink may be sacrificial, for the production of printed objects based on the bioink used that are removed from suspension baths or for the production of objects with open features (e.g. channels) where the ink is sacrificial and the bath is crosslinked for stabilization.

As bioprinting, and hence suspension bath bioprinting, has become more widely adopted in tissue engineering labs, a range of biological applications have been studied in the generation of both *in vitro* models and tissues for transplantation. Synchronously, new biomaterials have been developed that exhibit desirable properties for use as baths and to control features such as crosslinking. More recently, computational models have been developed to predict and analyze the properties of suspension baths and the bioinks that are extruded into them. With any developing field there are challenges around terminology, definitions, and other standardization issues. In this perspective, we first review the terminology used across the field and trends over the last decade before describing the specific characteristics of suspension baths and common bath materials. We then discuss how baths and printed structures are used before giving a perspective of the key challenges facing the field and future potential in suspension bath bioprinting. While not intended as a comprehensive review that covers all prior work, we hope that this perspective provides context to this rapidly growing field.

## 2. Literature and terminology

A key challenge when exploring and comparing the suspension bath bioprinting literature is the diversity of language used to describe the technique, print baths, and their associated features. Based on the definitions of **bioprinting**, ‘the use of computer aided transfer processes for patterning and assembly of living and non-living materials with a prescribed 2D or 3D organization to produce bio-engineered structures serving in regenerative medicine, pharmacokinetics, and basic cell biology studies’, **bioink** ‘a formulation of material(s) and biological molecules or cells processed using bioprinting technologies’, and **biofabrication** ‘the automated generation of biologically functional products with structural organization from living cells, bioactive molecules, biomaterials, cell aggregates such as micro-tissues, or hybrid cell-material constructs, through Bioprinting or Bioassembly and subsequent tissue maturation processes’, we performed a search with the aim of capturing the suspension bath bioprinting literature [18].

The query: ‘((ALL = (bioprint* OR print* OR writ*) AND ALL = (cell* OR tissue* OR biocompatib*)) AND TS = (vascular* OR embed* OR freeform OR suspen* OR granular OR microgel* OR support* OR slurry OR guest-host OR omnidirectional OR sacrific* OR fresh) NOT TS = (photovoltaic OR ‘liquid crystal*’ OR ‘fuel cell*’))’ was entered into the Web of Science database. The search was limited to articles published between January 2010 and December 2024 (inclusive), excluding review articles of which there have been many. Of the 19 452 articles that met these criteria, only 254 were determined to be relevant original research articles. Importantly, several prominent studies in the field were not captured in the search, highlighting the challenges in terminology used.

Across these 254 articles, over 50 unique descriptors for the method were identified (table 1). While some of them are highly specific, such as ‘Gelation of Uniform Interfacial Diffusant in Embedded 3D Printing (GUIDE-3DP)’, there was repetitive use of the terms ‘embedded’, ‘suspension/suspended’, ‘freeform’, and ‘sacrificial’.

**Table 1.** A list of descriptors that have been applied to suspension bath bioprinting approaches, as well as their frequency (*f*) and a representative literature reference that utilizes the term.

• 3D printing into/within…, *f* = 4, [6]
• Aspiration assisted freeform bioprinting (AAFB), *f* = 3, [19]
• Aqueous in aqueous printing, *f* = 1, [20]
• Biodot printing, *f* = 1, [20]
• Bioplotting, *f* = 1, [21]
• Bioprinting assisted tissue emergence (BATE), *f* = 1, [22]
• Constructs laid in agarose slurry suspension (CLASS), *f* = 1, [23]
• Direct ink writing, *f* = 1, [24]
• Embedded … [bioprinting, printing, direct ink writing, extrusion volume printing], *f* = 77, [25]
• Free directional printing, *f* = 1, [26]
• Freeform … [reconfigurable embedded all-liquid printing (FREAL), printing, reversible embedding, reversible embedding of suspended hydrogels (FRESH), freeze-FRESH], *f* = 58, [13]
• Gel-in-gel printing, *f* = 3, [27]
• Granular printing, *f* = 4, [28]
• Guest-host writing (Ghost), *f* = 1, [11]
• Human-based nanocomposite bioink printing (HUink), *f* = 1, [29]
• Immersion printing, *f* = 2, [30]
• In bath/gel printing, *f* = 6, [31]
• Infiltration-induced suspension printing, *f* = 1, [32]
• Intra-embedded printing, *f* = 1, [33]
• Liquid-in-gel printing, *f* = 1, [34]
• Low viscosity ink printing (LoV3D), *f* = 1, [35]
• Matrix assisted printing, *f* = 2, [36]
• Microgel assisted, *f* = 3, [37]
• Nanoclay suspension enabled printing, *f* = 1, [38]
• Omnidirectional printing, *f* = 6, [39]
• On-orbit printing, *f* = 1, [40]
• (Bio)printing into … [bath, fluid, granular, liquid-like support, matrix, microgel, support, suspended hydrogel], *f* = 13, [41]
• Print then … [lock, gel, solidify], *f* = 2, [15]
• Sacrificial printing, *f* = 4, [42]
• Sacrificial writing into functional tissues (SWIFT), *f* = 1, [43]
• Sequential printing in a reversible ink template (SPIRIT), *f* = 2, [44]
• Submerged printing, *f* = 2, [9]
• Support bath/matrix/material (assisted) printing, *f* = 32, [45]
• Support-less 3D printing, *f* = 1, [46]
• Suspended/suspension… [layer additive manufacturing (SLAM), manufacturing, printing], *f* = 11, [47]
• Writing in granular media, *f* = 1, [12]

The two most common terms identified were embedded (77 instances) printing and FRESH (58 instances). As mentioned, FRESH is a patented technique by Hinton *et al* that has been adopted by numerous studies across a variety of research groups [13]. Maloney *et al* used the term immersion bioprinting to describe printing tumor organoids into a gelatin bath for drug screening [48]. Submerged printing was used by Blaeser *et al* to create a range of hollow structures by printing alginate and agarose droplets into a fluorocarbon liquid [9]. Others refer to specific techniques with certain materials or applications such as ghost writing, while embedded and suspension or suspended bioprinting have been used as catch-all terms to describe printing into another material.

Along with the printing process, there are different terms used to describe the bath itself (table 2). There is less variability here compared to the descriptors for printing techniques, but the most common found term was ‘support’. Again, some terminology is more general than others, such as hydrogel and liquid support, microgel bath, and suspension bath, but some are very specific to a particular application. Kajtez *et al* uses ‘SHAPE’ to describe a bath made of annealable hydrogel microparticles based on alginate [49].

**Table 2.** A list of descriptors for suspension baths, as well as a representative literature reference that utilizes the term.

• ECM scaffold [50]
• Granular gel bath [51]
• Hydrogel matrix/support [52]
• Liquid bath/support [53]
• Liquid-like solid support [6]
• Microgel bath [17]
• Pre-gel bath [54]
• Self-healing annealable particle-ECM (SHAPE) [49]
• Self-healing hydrogels [11]
• Support … [bath, gel, hydrogel, material, matrix, medium, structure] [55]
• Suspension bath [56]
• Microporogen structured matrix [57]
• Yield stress… [bath, gel, matrix, support] [19]

## 3. Applications of suspension bath bioprinting

**Figure 3.**
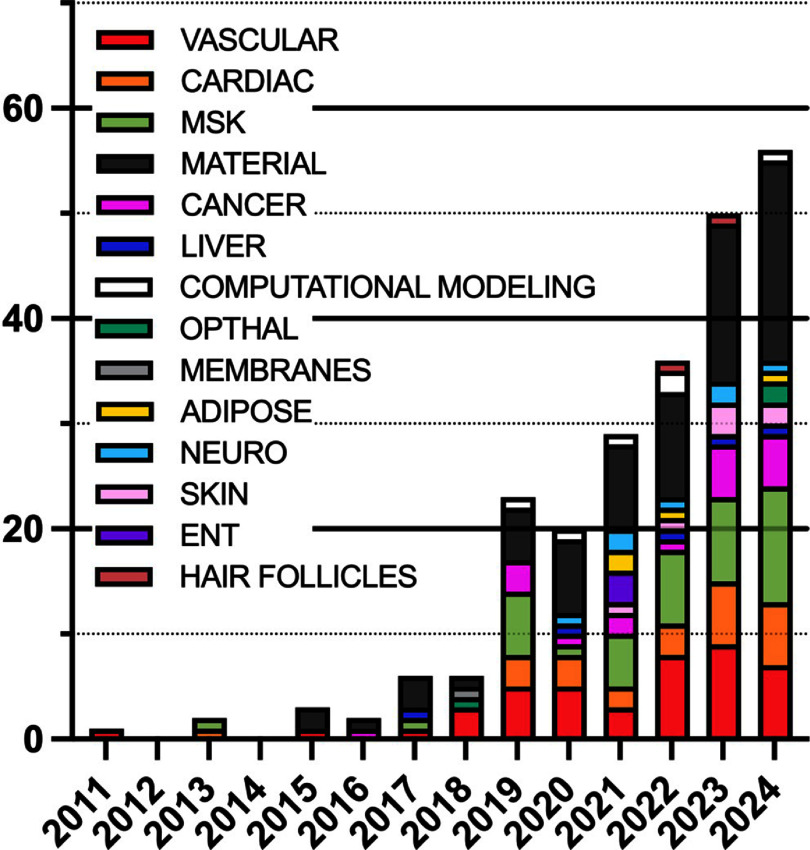
Distribution of suspension bath bioprinting topical areas from 2011-2024. Suspension baths have been applied to a wide variety of research areas. Vascular: ranges from artery tissues to microvascular networks in engineered tissues; musculoskeletal (MSK): includes bone, cartilage, meniscus and muscle; ENT: applies to ear, nose, and throat; Material: refers to new material developments without a distinct biological application; computational modeling: refers to computational models without a distinct biological application.

**Figure 4.**
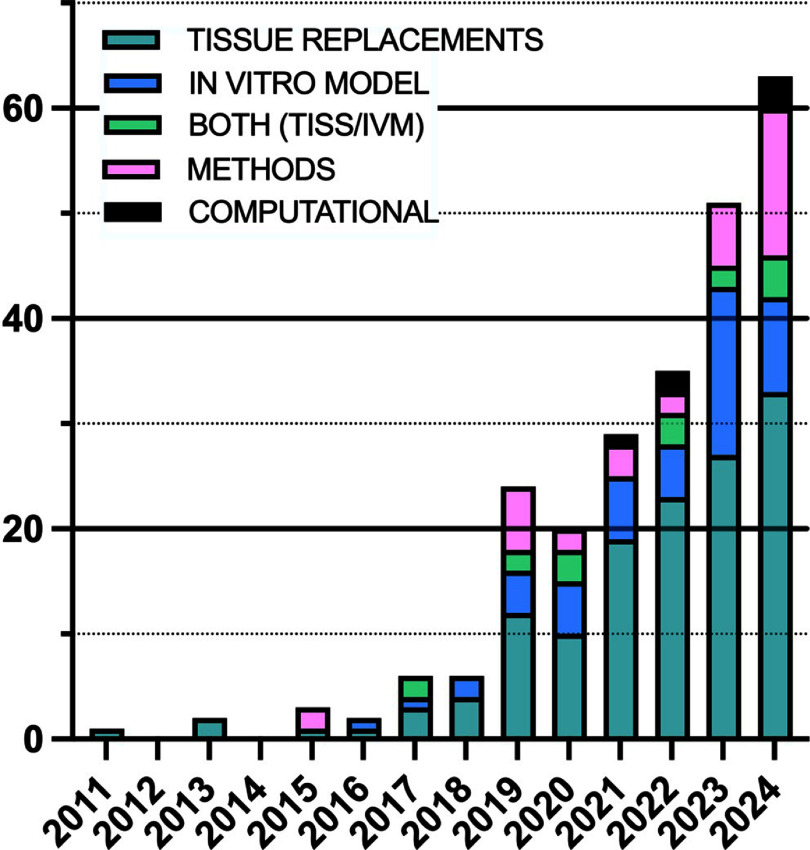
Distribution of suspension bath bioprinting applications from 2011-2024. Suspension bath printing has been applied to the engineering of tissues for use as therapeutic tissue replacements or *in vitro* models. ‘Both (TISS/IVM)’ refers to studies where their technologies are applied across tissue replacements and *in vitro* models.

Suspension bath printing has been employed for various biomedical applications as well as in food production, plant engineering and soft robotics. From the 254 articles that met our search criteria, we also observed trends in their applications and clinical targets (figures 3 and 4). The number of articles published every year has gradually increased (with an exception in 2020, likely due to laboratory shutdowns during the COVID-19 pandemic) with a growing range of applications being studied. A small number of papers have also focused solely on computational modeling, as shown in figure 3.

Taking a closer look at the distribution of topical areas where suspension bath bioprinting has been used in the exploration of both transplantable tissues and *in vitro* models, musculoskeletal (MSK) and vascularization are the most common behind studies that focused solely on material innovations. MSK is a particularly common application of tissue engineering and, within our search results, most studies focusing on MSK in suspension bath bioprinting were intended to produce implantable tissues, although *in vitro* modeling applications are gaining momentum in this area. Vascularization is an area that has been specifically targeted for suspension bath bioprinting as other bioprinting modalities struggle to fabricate tubular, branching structures that replicate the geometric complexity of microvasculature.

With regards to applications, studies can be grouped based on their investigation as tissue replacements, *in vitro* models, or solely new bioprinting materials/methods. It is interesting to note how the proportion of studies employing suspension bath bioprinting methods for *in vitro* disease models has increased over time relative to the development of potentially implantable tissues (figure 4). In the case of vascularization, 56% of these studies were intended as whole, or components of, tissue replacements, 18% intended as *in vitro* models, a further 22% stating that they could be used as either, and the remaining 4% of studies demonstrating computational modeling approaches [58, 59]. In cancer and neurological research, all studies were intended as *in vitro* models, while 83% of cardiac research articles were intended as tissue replacements over *in vitro* models. When taking a closer look at the studies that developed tissues, relatively few had employed their printed tissue mimics into animals to investigate integration, compatibility, or function. This is something we anticipate seeing more of in the coming decade as technologies continue to advance and tissue replacement strategies become more viable.

## 4. Bath structures and materials

As mentioned, suspension baths must be shear-thinning to enable the translation of a needle and deposition of material, as well as exhibit rapid recovery following shear yielding. While a specific range of values has not been defined, the recovery time following yielding must be sufficiently fast to prevent excessive flow that results in the deformation of the printed filament, but not so fast that the ink cannot form a continuous filament. A few studies have compared different suspension baths with recovery times between milliseconds and tens of seconds [47]. This is not a one-size-fits-all-solution, as there is a balance between the properties of the ink and bath to ensure adequate printing.

**Figure 5.**
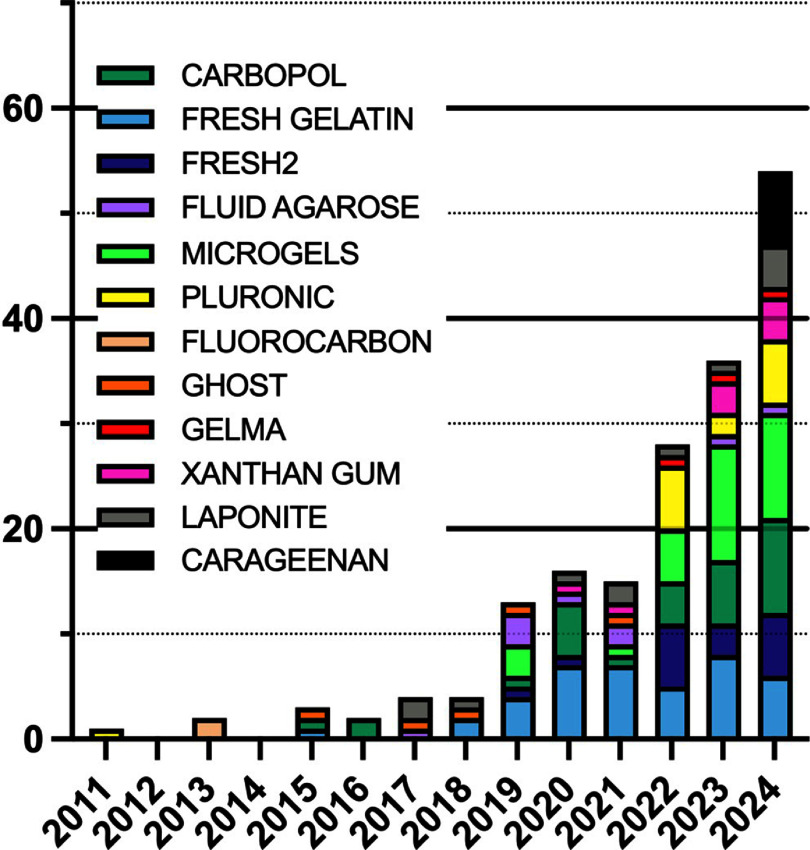
Prevalence of suspension bath types from 2011–2024. A range of baths have been explored in suspension bath printing. Here, the graph displays the most common and reused suspension baths. ‘Microgels’ covers a range of granular baths including fractured slurries (non-FRESH) and spherical microgels. FRESH2 refers to the second iteration of FRESH that uses coacervated gelatin.

Bath structure can be broadly defined in one of three groups [1]: viscous fluids [2], granular baths, and [3] suspensions (figure 5). ‘Viscous fluids’ include materials such as Pluronics [60, 61] fluorocarbons, collagen and gelatin derivatives [22, 62], xanthan gum [33, 63], and biopolymers [32, 64]. A key advantage of fluids as suspension baths is that beyond the initial material synthesis, no further structuring or processing is required. Another advantage is that high resolution is possible, which can be challenging when particulate systems are used. Considerations around using fluids as print baths are that the ink and bath material may mix if the surface tensions are not sufficiently dissimilar. Similarly, the contributions of surface tension can affect print fidelity as a result of drag in the print bath. Several groups have also developed aqueous 2-phase systems that consist of different hydrophilic polymers with very low interfacial tension. In these systems, the interface between the bath and ink allows the rapid transport of biomolecules such as proteins and nucleic acids [65].

Guest-host hydrogels, composed of supramolecular polymers based on guest-host interactions can also be considered as a fluid bath. These materials have non-covalent interactions that allow for easy disruption and thixotropic properties, rendering them injectable and with fast self-healing properties. Highley *et al* and Song *et al* used these guest-host gels as baths, with the later study developing perfusible vessels to study angiogenesis by crosslinking the bath after printing [11, 52]. One of the challenges of these materials is the complex synthesis involved, which can limit scale-up. Further, controlling the specific properties of the material can be difficult as the modification and interaction of each polymer must be very precise.

**Figure 6.**
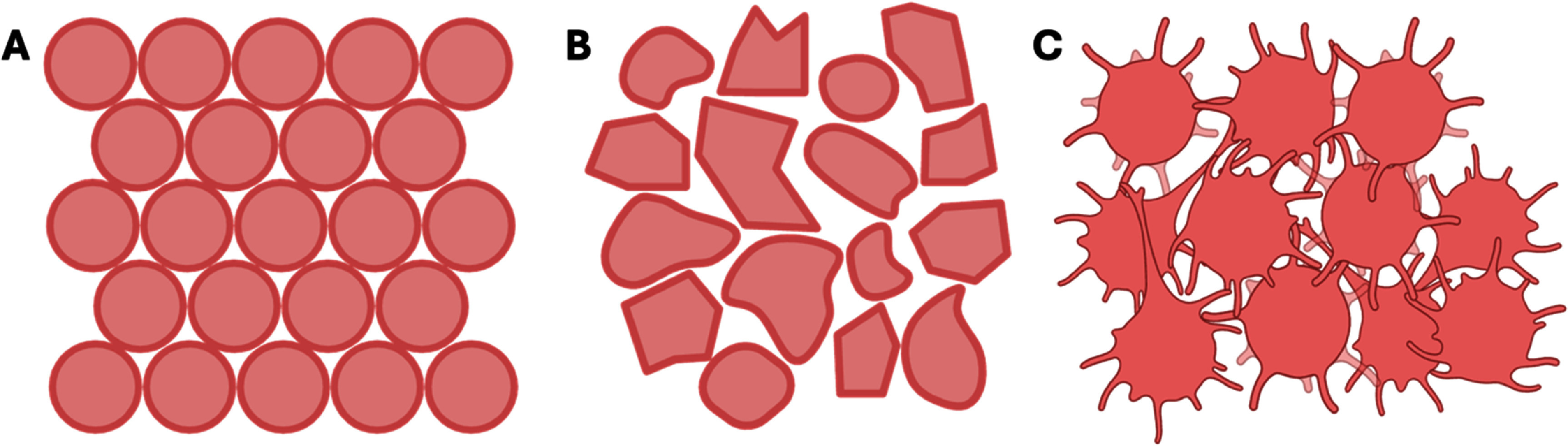
Schematic of granular bath structures. (A) Spherical particles; (B) fragmented particles; (C) fluid gel.

‘Granular’ baths encompass the following terminologies for baths: granular, microgels, fluid gels, slurries and fractured gels to name a few, and were the most commonly used suspension baths identified in our literature search. These baths are made up of hydrogel particles that range in size from <1 to 500 *µ*m in diameter, existing in some type of interstitial matrix or fluid. Particles are commonly formed through microfluidic dropletting, batch emulsion processing, or coacervation to form spherical particles, fragmentation to form irregular particles, or by agitation during gelation to form fluid gels (figure 6). The most prevalent are fragmented slurries, formed by blending or homogenizing an already crosslinked gel, resulting in irregularly shaped particles [13]. Silberman *et al* used fractured particles made of sodium alginate, sodium chloride, and xanthan gum into which vascularized cardiac tissue constructs were printed [66]. One disadvantage is that the shape of these particles cannot be controlled, which can decrease print fidelity. It is relatively easy, however, to control their size by increasing the blending or homogenization time. More control over fidelity can be gained by producing spherical microgel particles. An example of this was in the shift from FRESH to FRESH2 (commercially known as life support) by the Feinberg lab. They progressed from blended irregular-shaped particles to coacervated spherical particles, which led to increased control over gelatin particle size and shape, resulting in more reproducible print baths and better print resolution. Collagen solutions were printed into this bath in the form of capillaries and a scaled human heart showed that individual fiber resolution was increased by a factor of ten (from 200 *μ*m to 20 *μ*m fiber diameter) when compared to the original method [67]. This technique has also been used in many other instances. For example, De Santis *et al* used the FRESH method to print alginate and extracellular matrix (ECM) to create bioprinted human airways with epithelial progenitor and smooth muscle cells [68]. Flégeau *et al* used this method for auricular cartilage engineering by printing hyaluronic acid-tyramine microgels [69]. Batch emulsion and microfluidics are other common fabrication methods for uniformly spherical particles, the former being much faster but with a larger size distribution of particles. Molley *et al* [70] used batch emulsion and Mo *et al* [40] used microfluidics to create spherical particles for their suspension baths.

Muir *et al* characterized various ways to make microparticles, including extrusion fragmentation, batch emulsion, and microfluidics from norbornene-modified hyaluronic acid [71]. They found that composition and method of fabrication greatly affected viscosity, storage modulus, pore size and void space volume of the jammed granular material. For example, microfluidics produced the most uniform spherical particles with a narrow size distribution, batch emulsion produced spherical particles but with a greater distribution, and fragmentation produced the most irregular and widely distributed particles. The throughput of these approaches should also be considered, as methods like fragmentation and batch emulsion can produce large amounts of particles rapidly, whereas microfluidics may be slower. This understanding of particle production and properties aids in the understanding of different bath structures and which approach may be the most appropriate for different applications. A disadvantage of granular baths is the potential for residues within the printed parts if particles become entrapped in the crosslinked structure. Further, for very high resolution printing, surface imperfections in filaments have been observed that are not seen in viscous liquid baths.

Fluid gels are a type of granular gel since they exist as hydrogel particles within an interstitial fluid. A key difference is that they are generally irregularly shaped, with agarose forming a ‘hairy’ particle morphology and gellan gum forming ribbon-like structures [72]. The most common route to their fabrication is with thermogelling polymers, but they can also be formed with ionic crosslinking mechanisms. With the application of shear during crosslinking, usually through stirring, individual gel particles are formed. Senior *et al* [47] used this method to fabricate agarose as a fluid gel bath, showing that their size and shape can be controlled by modulating the applied shear and polymer concentration, whereas Compaan *et al* [16] used gellan gum as the bath. Mendes *et al* used the addition of low concentration (11 mM) calcium chloride to agarose fluid gels to stabilize ionically crosslinked inks immediately after printing [29]. A key advantage of a fluid gel system is the ease of fabrication of large volumes of bath material as well as the relatively low cost of the raw bath materials. A disadvantage is the limited control over particle shape, which may limit the printing of very small features and the potential for bleeding of very low viscosity inks.

Retrieving printed constructs from suspension baths is another challenge in the field, which usually involves gently removing excess bath material mechanically or with washing, which may not be appropriate for very delicate printed structures. Printed constructs may also contain residual particles, which may not be desired in final printed constructs and should be considered in the washing process. Thus, there is a balance between the rigor of washing to remove bath components and the stability of what are often delicate structures. This should also be considered with regards to the ink and bath compatibility, which has been recently investigated across a range of materials with respect to interfacial stabilities [73]. Lastly, the use of crosslinkers is needed to stabilize many materials and the washing process should consider removal of potential toxic components during printed construct retrieval.

As one interesting granular bath, Skylar-Scott *et al* developed the technique ‘sacrificial writing into functional tissue’ (SWIFT) whereby hundreds of thousands of cell aggregates or organoids, termed organ building blocks, were jammed in an ECM-based interstitial matrix, to form a suspension bath [43]. Sacrificial ink materials were then extruded into these cell-dense baths to create lumen-like channels. The baths exhibited all of the typical suspension bath rheological features and they showed very good control of filament morphology (lumen diameter) by changing print speed. While this technique may be challenging for high throughput production of tissues, it is a great example of how cells can be used as the bath, rather than only as an ink. This approach has been recently expanded with co-SWIFT, where coaxial sacrificial writing is used during the printing process to introduce smooth muscle laden vessels into printed constructs [74]. Additional methods have been investigated to include cells into suspension bath printing, such as with ‘sequential printing into a reversible ink template’ (SPIRIT), which includes a microgel-based biphasic material as both a bioink and bath that can encapsulate cells [44].

Another class of baths, that behave similarly to viscous fluids, are ‘suspensions’. Suspensions contain small solid objects uniformly mixed in a fluid. This includes things such as (nano)fibers, individual cells, and nanoclays (usually laponite). Because the footprints of nanoscale materials on printed filaments are much smaller compared to granular baths, a particular advantage of such support materials based on nanoparticle suspensions is the high resolution of printed structures. Shin *et al* used a suspension of hydrophilic and hydrophobic cellulose nanofibers (CNF) to produce a microfluidic cell culture device that has selective diffusion depending on the CNFs present [36]. Jin *et al* used a nanoclay suspension bath to print thermosensitive (and ionically crosslinkable) materials, which can be difficult with competing thermosensitive baths such as gelatin [15]. One strength of the suspension class is the possibility for dual properties. For example, Bilici *et al* used a bath that was a nanoclay suspension in Pluronic with sodium bisulfite added as a crosslinker [75]. Additionally, Bakht *et al* developed a bath made of cellulose nanocrystals, which not only enabled bioprinting constructs with high filament resolution but was also easily self-assembled into a fibrillar material by addition of calcium ions post-printing, functioning either as a tailored bioreactor for the long-term *in vitro* maturation of printed constructs or to directly fabricate perfusable organs-on-chip [76]. The printing detail achieved with this type of support bath further enables other unconventional concepts, such as magnetic assisted bioprinting, where the orientation of magnetic responsive microfibers within hydrogel bioinks can be controlled during printing without the structural disturbances observed with other baths, such as fluid gels [77].

## 5. Bath and print characterization

Suspension baths can be characterized based on their structure, rheology, print fidelity, and other factors. Increasingly, these have been computationally modeled to predict print outcomes and to develop tools for bath and ink selection.

### 5.1. Rheological characterization

**Figure 7.**
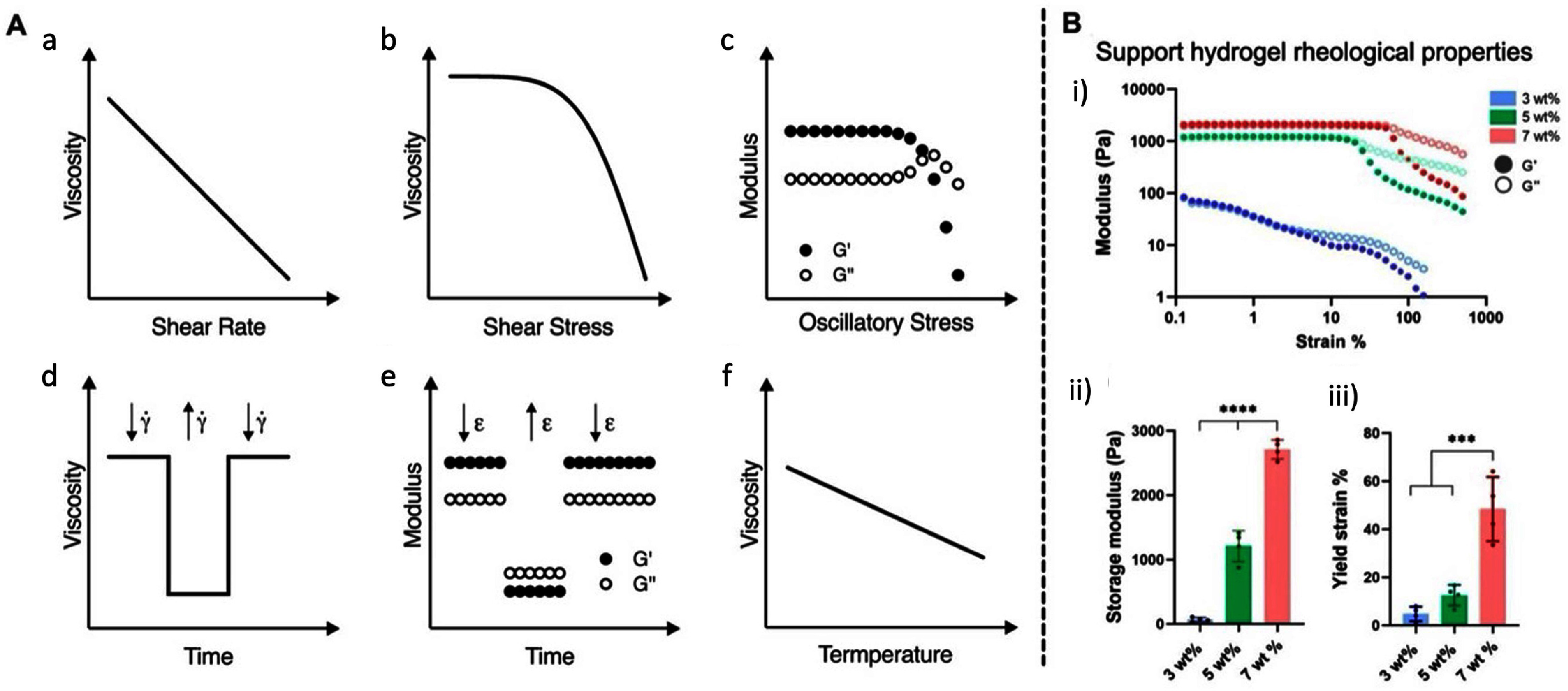
Examples of rheological tests for suspension bath printing. (A) Classic profiles obtained from rheological tests: (a) shear rate sweep, (b) stress ramp, (c) oscillatory stress ramp (amplitude sweep), (d) rotational thixotropy (changing shear rate, $\mathop \gamma \limits^ \cdot $), (e) oscillatory thixotropy (changing oscillatory strain, ϵ), and (f) rotational temperature sweep. Reproduced from [78]. CC BY 4.0. (B) Support hydrogel rheological properties: (i) oscillatory strain sweep of 3 (blue), 5 (green) and 7 (red) wt% guest-host hydrogel, (ii) moduli and (iii) yield strain extracted from strain sweep (i). Reproduced from [79]. CC BY 4.0.

Rheology is the most performed characterization method for both suspension baths and bioinks. General parameters for suspension baths are: a clearly defined yield-stress behavior, shear-thinning flow profiles, and recovery from shear stress in an anti-thixotropic manner. It is important to note that some fluid baths exhibit Newtonian behavior. Different rheological tests can shed light on these properties; classic profiles and example data are shown in figure 7(A). The yield-stress or strain indicates when the suspension bath material yields. It is commonly determined by an oscillatory stress or strain sweep, where increasing stress or strain is applied and the yielding behavior is defined as when there is deviation (reduction) of the storage modulus (G’) from baseline. This test indicates the force required for the material to be deformed by a moving needle or displaced by extruded material ink. Shear-thinning describes a material whose viscosity decreases as increasing shear forces are applied. This is commonly determined by a flow shear rate sweep. A sweep of increasing shear rates are applied to the material while viscosity is measured; in shear-thinning materials, there is a gradual reduction in viscosity with increasing shear rate. Self-healing properties, which indicate if a material can be deformed and then recover to its initial state, are often tested by a 3- or 5-step thixotropic test. In this experimental procedure, alternating low and high strains are applied, mimicking the application and removal of forces to a suspension bath as a needle is translated through it. We refer the reader to a detailed review of rheological analyses in suspension bath bioprinting by Cooke and Rosenzweig [78]. As a specific example of rheological data for a suspension bath, Daly *et al* measured the rheological properties of a bath from guest-host hyaluronic acid hydrogels at various polymer concentrations (figure 7(B)) [79].

These specific rheological properties of the bath enable the printing of low viscosity and soft inks, one of the key advantages to suspension bath printing. Some common inks used are Pluronic [80, 81], cell laden materials [82, 83], and naturally occurring biomaterials such as modified gelatin, hyaluronic acid, alginate, ECM, and other biological components [25, 84]. For example, Becker *et al* [35] used multiple non-viscous inks such as alginic acid, gelatin, and heparin into a poly(ethylene glycol) bath, with values as low as 4 mPa. These inks could not be printed into complex, porous structures without the use of a suspension bath. There is currently no standardization around rheological testing regarding experimental setups or testing parameters, which makes it somewhat difficult to compare and select an ideal bath for a certain outcome.

Yogeshwaran *et al* recently provided a detailed rheological analysis of fractured gelatin and agarose slurries; by changing the blending time they achieved different sized particles. With increased blending time smaller particles were obtained, which led to shear-thinning behavior at much lower shear rates compared to larger particles (formed with shorter blending times) [85]. Rheological data can also be used to inform the fidelity of suspension bioprinting; Friedrich *et al* found that in water-based systems, filament shape is controlled by the local viscosity ratio near the nozzle [86]. Interfacial tension between ink and bath is also a key factor in filament surface roughness; non-zero interfacial tension gives rise to much rounder, smoother filaments than ink-bath combinations at zero interfacial tension [86].

### 5.2. Bath structure and deformation

In the initial assessment of granular baths, a common technique is to image the particles and determine the size, shape and bath porosity. Optical clarity may also be desired in some applications to both see the print as well as for particle imaging. For example, Machour *et al* found that their particles were spherical and with an average diameter of 21 *µ*m. They used qualitative imaging techniques to determine their bath was optically transparent and then used fluorescence recovery after photobleaching to study diffusion within it [87]. Work by the Feinberg lab used optical coherence tomography (OCT) to image internal details of structures printed into FRESH baths [88]. They were able to image both during and post-printing through the bath, which was improved using a refractive index matching solution (iodixanol) that rendered the gelatin microparticle bath optically clear. Both collagen and cell-seeded fibrin scaffolds were imaged using the method, giving rationale for the production of optically clear suspension baths to verify print fidelity during printing [88].

Particle imaging velocimetry around the print nozzle has been performed in a nanoclay (laponite) bath to assess the deformation of the bath; by changing the volume fraction of the nanoclay suspension, the structural breakdown and orientation times were very different. Further, when compared to a Carbopol bath, the thixotropic length scale of nanoclays was shorter due to the nanoscale structure of laponite particles [15].

### 5.3. Biological outcomes

With the greater use of cellular components within printing processes, including high cell density inks and baths, a greater assessment of cell behavior during and after printing is needed. For example, there are many features within bioinks and baths that may influence cells, such as the temperature and pH during printing, as well as the diffusion of reactive components and even residual bath components within prints. There has been a very limited focus on cell behaviors within these platforms and no systematic study regarding bath and ink properties, although much can be gained regarding prior studies on extrusion printing of bioinks and cellular behaviors [89, 90].

Often, cells are characterized only for their viability, which is important, but the success of printed constructs is dependent on cell function beyond whether they survive the printing process. For example, in tissue engineering, other outcomes such as cellular motility and proliferation are important, as well as outcomes such as cellular differentiation and matrix production. These are highly dependent on the surrounding microenvironment of the cells [91]. Depending on the application, additional consideration to these outcomes should be monitored in printed constructs.

### 5.4. Measuring the fidelity of prints

Print fidelity is an important parameter in any type of 3D printing and it refers to how closely the printed construct matches the theoretical expectation, usually the CAD model. Methods include dimensional analysis of geometric features [92], mathematical model comparisons [93], fiber morphology [94], mathematical calculations of parameters such as printability [87], and test prints [95]. The most common way to fine-tune print fidelity is adjustment of printing parameters such as print speed and extrusion or flow rate. Secondary to this, studies have changed the concentration of polymers in both the ink and bath. Most characterization techniques to aid in the analysis of fidelity are based around imaging, such as, brightfield, confocal, computed tomography, and others to assess fiber morphology and test prints. For example, Bliley *et al* used both brightfield and OCT to analyze the fidelity of the prints [96].

**Figure 8.**
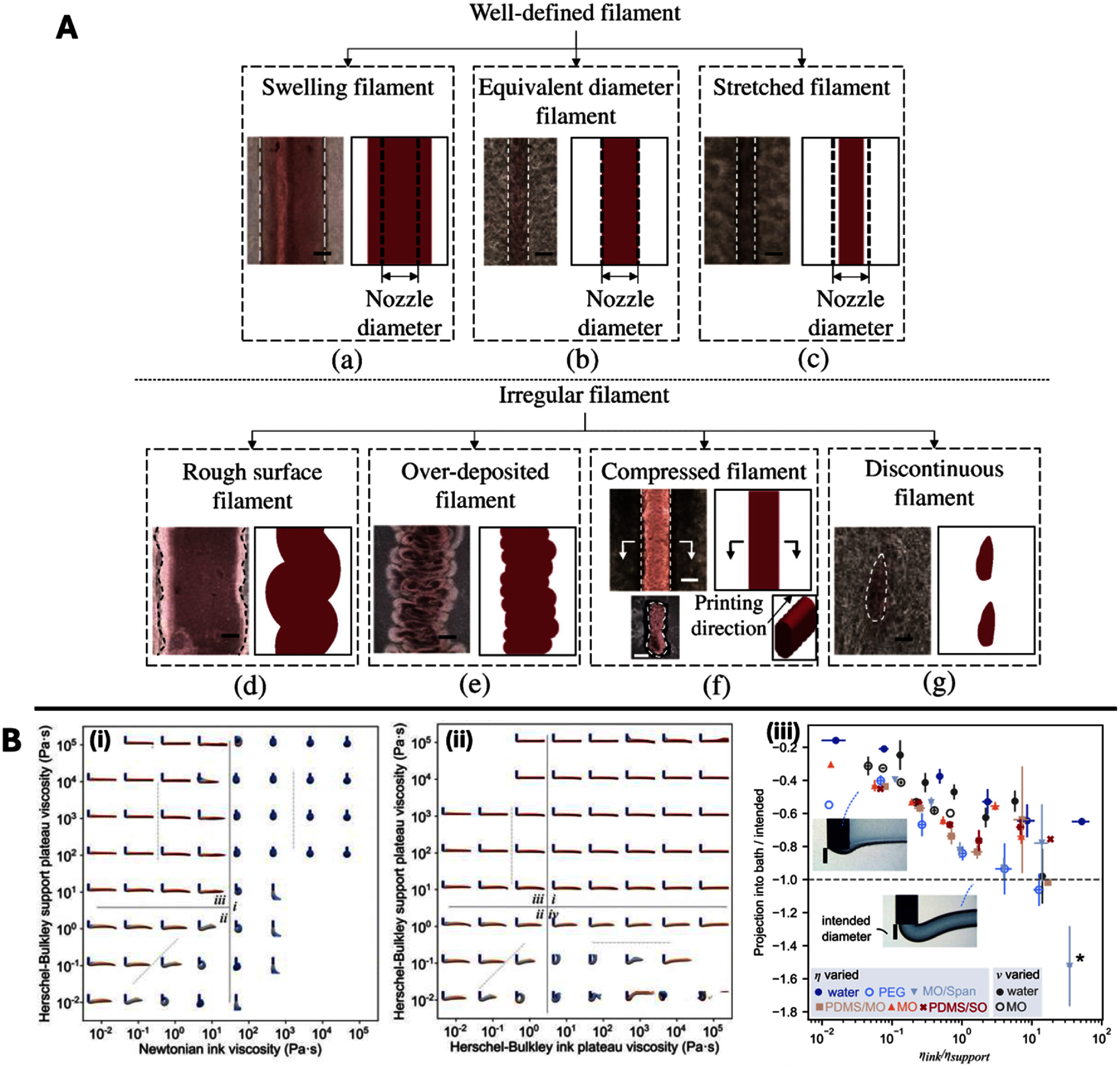
Examples of filament morphology fidelity tests in suspension bath printing. (A) Top-down views of filaments of alginate-gelatin ink printed into a laponite nanoclay bath. Reprinted with permission from [15]. Copyright (2017) American Chemical Society. (B) Side-view of modeled filaments of (i) Newtonian or (ii) Herschel–Bulkley inks, deposited into Herschel–Bulkley supports, (iii) experimental data investigating the viscosity ratio of bath/ink and vertical displacement of the printed filament. Reprinted with permission from [86]. Copyright (2022) American Chemical Society.

Filament morphology is an important basic fidelity parameter to ensure that subsequent prints reach high geometrical similarity to the designed part and there are numerous factors that can be considered. Jin *et al* presented 7 different filament shapes that are observed with different print parameters in a nanoclay bath (figure 8(A)) [15], while Prendergast and Burdick showed how print speed and bath polymer concentration affected filament deposition both theoretically with structural similarity index measure and experimentally with fluorescently labeled inks in a fluid agarose or Carbopol bath [97]. Friedrich *et al* published extensive work modeling filament shapes, as well as investigated the challenges of shear stresses applied to inks during printing [24, 98, 99]. As part of their work on filament morphology they investigated the compatibility of baths and inks with similar or contrasting rheological properties (and surface tensions) regarding filament cross-sectional area, aspect ratio, and any curling or deformation that affected vertical (*z*-plane) positioning or horizontal (*x–y* plane) fidelity. An example is shown in figure 8(B)(i, ii), where the side-plane of modeled Herschel–Bulkley or Newtonian ink filaments one second after extrusion into a Herschel–Bulkley suspension bath is shown. Their recommendations and results stand as an incredibly helpful design tool as researchers select ink-bath pairings for desired filament shapes. Considering vertical positioning, a commonly observed effect in suspension bath bioprinting is the filament rising above the nozzle following deposition. If this is not considered during printing, there is a high chance of the needle dragging through previously deposited material in multi-layer suspension bath printing. In their 2022 work, the same group showed that with increasing viscosity ratio (viscous inks printed into low-viscosity baths), printed filaments were projected lower into the bath, and vice versa (figure 8(B)(iii)) [86]. On a smaller scale, the analysis of surface imperfections has been studied in a comparison between granular and viscous liquid-type baths, with the former often leaving bumps or residues on filaments [45]. As noted above, some of these challenges are also overcome with the use of smaller components, such as nano-scale structures within baths.

At a larger scale, an elliptical window calibration is an example of a suspension bath print test whereby the optimization of bioink and printer settings should produce a perfect elliptical window with dimensional accuracy and no excess of material across the window or above it when printing is complete [100]. Similar to the challenges in rheological characterization, there is currently a lack of standardization across the field to replicate this test for new or existing suspension baths.

## 6. Challenges and future directions

With any developing field, there are challenges as techniques develop and expand. One of the largest issues is the lack of uniformity across the field, especially in the areas of terminology, characterization, and metrics of ‘success’. In the case of terminology, there are many variations for both the printing process and bath, which makes it challenging to find the appropriate literature. We advocate for the use of the term ‘suspension bath bioprinting’ to describe the technology described here. Characterization of this print process can also be challenging and there are many relevant techniques and attributes to consider, such as bath and ink properties, printing parameters, resolution, tool paths, and other goals of the technology. Characterization can be complex, and not every article uses the same characterization techniques. Some of these features may also be competing; thus, comprehensive characterization is an important consideration in 3D printing to ensure the best possible outcomes and contribution to the wider field. Related, metrics of success can vary greatly depending on application and goals, and there may be multiple metrics for every print and construct. Recent developments such as the integration of microscopy and computer vision for *in-situ* process monitoring during suspension bath bioprinting are now providing faster tools to assist in the rational decision making to enhance the reproducibility of printed constructs [101]. Overall, we advocate for a thorough characterization of inks, baths, and their interactions with any new suspension bath printing platform that is described.

Another recent advance in the field is the combination of suspension bath printing with other 3D printing modalities. For example, Ribezzi *et al* [5] and Riffe *et al* [102] showed the combination of suspension bath printing with volumetric additive manufacturing. This approach utilizes the benefits of both 3D printing techniques, allowing for more complex prints than either could achieve alone. There is the potential for many other advancements by continuing this trend of combining other manufacturing methods with suspension bath bioprinting, such as xolography [103]. We encourage approaches that interface suspension bath printing with new printing methods as they develop, which will allow for new levels of controls over inks at various scales to meet the challenges in tissue engineering ahead.

Another emerging trend is the use of this manufacturing method to produce prints with anisotropy. Li *et al* [27] and Prendergast *et al* [104] utilized suspension bath bioprinting to create anisotropic constructs for skeletal muscle and meniscus tissue, respectively. This approach allows for more complex and biologically relevant structures. Living organisms are also not static systems, and time dependent changes occur during development, morphogenesis, and mature adult function. Suspension bath bioprinting is also evolving into 4D bioprinting concepts that capture features of these time dependent functions, for example, on supporting programmed shape-morphing of printed tissues [105], or through user-controlled systems based on temperature [106], magnetic [77], or light triggered stimuli [107]. These are just a few examples that have been explored in this area, but the integration of dynamic approaches such as these will become increasingly important in the near future.

Suspension baths are also being used to print cell-only materials, primarily for *in vitro* modeling where the potential contributions of non-native materials can be eliminated from a drug screening or pathology-mimicking system [6]. This also allows for the fabrication of very high cell dense structures, which may better mimic the cellularity of tissues [43]. There is also exciting progress being made in the integration of suspension bath bioprinting with organoid technology. Lutolf’s team has pioneered this concept, proposing the use of bath materials to extrinsically control the patterning and morphogenesis of organoids to recapitulate complex macro-scale tissue architectures [22]. The cost of generating large numbers of organoids is likely a major barrier contributing to the slow adoption of this concept in the field. However, recent studies have pushed forward this line of research, proposing custom piezoelectric printheads that allow the fast and precise printing of organoid ‘seeds’ that grow and self-organize into different tissues within the supporting material [108]. The printing of cell only material is likely to be an important approach, which leverages the biological interactions of cells with each other to enhance cell-cell signaling and to limit the need for biomaterials themselves. As described above, challenges exist to ensure compatibility with the printing processes and cellular viability and other outcomes.

It is interesting to note other approaches that leverage aspects of suspension baths during printing, albeit without the typical bath and ink components. For example, these yield-stress materials have acted as reservoirs for the building of cellular components to generate tissue model systems. Specifically, Daly *et al* used a guest-host bath and micro-manipulator to precisely place cell aggregates of varying composition into 3D configurations to model cardiac fibrosis. The shear recovery properties of the bath allowed them to carefully place the aggregates into patterns and the aggregates then fused together into microtissue structures [79]. The Ozbolat lab used a dual-reservoir system in this approach, where one side consisted of a Carbopol suspension bath and the other a bath of cell culture media. Cell spheroids in the cell culture media were picked up by an aspiration system, moved across an interface and precisely deposited into the Carbopol bath to construct a range of complex structures, including double helical structures, that would not be possible to produce in air [19]. Such plug-and-place systems allows for the use of smaller numbers of cells with high specificity of spatial patterning.

Considering the bigger picture of this technology in the biomedical research space, the fastest way to clinical utility or impact of suspension bath bioprinting is likely in the generation of *in vitro* disease or personalized drug screening models. The regulatory environment makes the goal of implantable cell-seeded tissue constructs still very challenging and likely many years if not decades away. Some of the challenges ahead in clinical translation of transplantable constructs related to suspension bath printing include features such as high cell viability during printing, the stability and mechanical robustness of printed constructs, and the minimal contamination of printed constructs with residual bath materials. Despite these considerations, the significant efforts in the field have moved suspension bath bioprinting forward in the last decade and enable the promise of innovative strategies in the future that have real world impact on clinical patient outcomes.

## Data Availability

All data that support the findings of this study are included within the article (and any supplementary information files).
